# Has children’s oral health-related quality of life improved more following necrotic primary molars pulpectomy or extraction?

**DOI:** 10.1038/s41432-024-01020-8

**Published:** 2024-05-29

**Authors:** Majidi Bakar, Brett Duane

**Affiliations:** Dublin Dental University Hospital, Trinity College Dublin, Dublin, Ireland

**Keywords:** Dentistry, Dental public health

## Abstract

**Study design:**

A randomised parallel controlled clinical trial was conducted between 2013 and 2015 at the University of Sao Paulo, Brazil, to assess the impact of pulpectomy or extraction on the oral health-related quality of life (OHRQoL) of children with pulp necrosis in primary molars.

**Study selection:**

Children between the ages of 3 and 5 who were in good health but had extensive caries in at least one primary molar with signs of pulpal necrosis (also as seen radiographically, caries reaching the pulp with no signs of internal or external resorption) were considered for inclusion. Additionally, teeth with sufficient structure for rubber dam placement were also included. Children with any systemic, neurological, or other conditions that negatively impacted their growth were excluded.

**Clinical procedures and success criteria:**

After computer-generated randomisation, 100 children were assigned randomly into two groups: 50 in the pulpectomy group and 50 in the dental extraction group. A paediatric dentist performed all procedures under local anaesthesia without sedation or general anaesthesia, and a rubber dam was used for pulpectomy with composite restoration in a single session. The OHRQoL scores were evaluated at baseline, 4, 8, and 12 months using the Brazilian version of the Early Childhood Oral Health Impact Scale (B-ECOHIS) via face-to-face interviews with parents conducted by a researcher trained in a single-blinded fashion. Additionally, the child’s self-reported dental anxiety was measured using the Facial Image Scale (FIS), and dental pain was assessed using the Wong-Baker Faces Pain Scale (WBFPS) immediately after the treatment as secondary outcomes.

**Results:**

The mean difference (SD) in the total B-ECOHIS score between baseline and after 12 months was 12.66 (6.79) for the pulpectomy group and 10.94 (9.28) for the extraction group, with effect sizes of 3.2 (95% CI: 2.42–4.20) and 1.4 (95% CI: 0.84–2.11), respectively. While both treatments significantly improved the children’s OHRQoL after 12 months, the pulpectomy group showed greater long-term improvement compared to the extraction group, with mean differences (SD) of 4.86 (6.13) and effect sizes of 0.8 (0.46–1.13; p < 0.001). Moreover, children in the extraction group showed higher levels of anxiety compared with those in the pulpectomy group at 12-month follow-up (OR = 2.52; 95% CI = 1.30–4.89), and they reported 93% more odds of ‘dental pain with high level’ immediately after treatment than those in the pulpectomy group (OR = 1.93; 95% CI = 0.83–4.49).

**Conclusion:**

Children treated with pulpectomy in their necrotic primary molars were found to have better OHRQoL than those who had their primary molars extracted.

## A Commentary on

**Abanto J, Tsakos G, Olegário I C** ***et al****.*

Impact of pulpectomy versus tooth extraction in children’s oral health‐related quality of life: A randomised clinical trial. *Community Dent Oral Epidemiol* 2024; 10.1111/cdoe.12895.

**GRADE Rating**: 

## Commentary

Utilising a patient-reported outcome measure (PROM), such as the OHRQoL, dental anxiety, and pain reports, offers favourable alternative assessment methods for both endodontic treatment and dental extraction, as they assess treatments from the patient’s perspective. This addresses the challenges of evaluating endodontic treatment outcomes based solely on clinical and radiographic success^1^, which differ from dental extraction outcomes, such as those related to space loss^2^.

In this randomised clinical trial (RCT), the authors focused on assessing the impacts of treating primary molars with pulp necrosis (either through pulpectomy or extraction) on children’s OHRQoL^3^. The outcomes included the OHRQoL and dental anxiety scores assessed at baseline, 4, 8, and 12-month follow-up, along with dental pain scores measured immediately after each treatment. The inclusion and exclusion criteria are well-documented and appropriate.

Randomisation was carried out using a computer-generated procedure before the commencement of the dental procedure. Although the generated sequence was enclosed in opaque, sealed, and sequentially numbered envelopes without the participants’ knowledge, there was no mention of whether this allocation sequence was concealed from the researchers.

Participants were blinded to the intervention they received, and the allocated group was revealed only after inclusion, before the intervention. Since the procedures were performed by the same individual who conducted the research, there is a potential for performance bias, as this knowledge may inadvertently influence the way the procedure was carried out. A separately trained researcher who administered the B-ECOHIS at baseline, 4, 8, and 12 months was unaware of the children’s clinical conditions before randomisation into the treatment group.

Participant baseline characteristics, including sex, age, household structure, household income and caries experience, were clearly defined for each study group to avoid potential selection bias. However, there were variations in the proportion of females to males between the pulpectomy and extraction groups. This is potentially problematic as one gender could be more susceptible to dental anxiety or perceive pain differently, which could influence their reported OHRQoL, potentially introduce bias into the results and make it challenging to apply the results of the study to a local population.

Both pulpectomy and extraction procedures were thoroughly described following recommended clinical guidelines^4,5^. it would have been good to have included the significance level and power in the sample size calculation. A CONSORT flowchart was included, providing relevant participant information flow throughout the RCT with consistent follow-up intervals for each study group. The authors employed an intention-to-treat analysis, incorporating conditional multiple imputation to address missing data resulting from the loss of follow-up in 12 out of 100 children by the 12-month follow-up.

The authors discussed the main findings of the study^3^: Children who had pulpectomy experienced five times greater improvement in long-term OHRQoL compared to those who had extractions and greater effect size (0.8, 95% CI: 0.46–1.13; p < 0.001). Meanwhile, children in the extraction group were 2.5 times more likely to have higher levels of anxiety compared to those in the pulpectomy group after 12-month follow-up (OR = 2.52; 95% CI = 1.30–4.89). Additionally, children in the extraction group were two times more likely to experience ‘dental pain with high level’ immediately after treatment than those in the pulpectomy group (OR = 1.93; 95% CI = 0.83–4.49). However, the wide confidence interval for both secondary outcomes suggest uncertainty about the exact magnitude of this difference. This indicates that it is difficult to be confident about the true odds ratio. A larger sample size may be needed to narrow down this range and provide a more precise estimate of the difference in anxiety levels and dental pain between the two groups.

Although the authors effectively presented the main study findings in table format for total B-ECOHIS and dental anxiety scores, there were no tabulated results for dental pain^3^. This absence may indicate incomplete reporting of study findings, raising concerns about transparency and the reliability of the research. The authors only provided estimates of the odds ratio between the intervention groups regarding pain levels (OR = 1.93; 95% CI = 0.83–4.49). It also would have been good to include p-values, as it can be challenging to determine if the observed results are due to chance or if they reflect a true effect.

The B-ECHOIS is a validated tool in the literature for evaluating the influence of oral conditions on children’s quality of life^6^. Utilising the OHRQoL assessment as the primary outcome of this trial signifies a shift from traditional tooth-oriented criteria to an evaluation that considers both patients’ and parents’ experiences when deciding on the most appropriate treatment plan^7^. The study could have improved by ensuring a balanced distribution of participants’ gender across each intervention group during the randomisation process. Additionally, focusing on a single age group, such as a 3-year-old group only, could have enhanced the study, as combining different age groups may yield varied results; older children typically show a lower negative impact on the OHRQoL compared to younger children. The authors did not provide comprehensive information on all the complications or side effects of primary molar pulpectomies and extractions. Furthermore, there needed to be a discussion on the cost-effectiveness of both interventions.

In conclusion, this study contributes valuable evidence supporting pulpectomy over extraction for treating necrotic primary molars in children. However, careful consideration of its limitations and further research is required for broader applicability.

Practice point

- When treating primary molars with pulp necrosis, a better OHRQoL can be achieved with pulpectomy than extraction. However, the limited available evidence requires careful interpretation.
